# Medicinal Cannabis Prescribing in Australia: An Analysis of Trends Over the First Five Years

**DOI:** 10.3389/fphar.2022.885655

**Published:** 2022-05-10

**Authors:** Sara L. MacPhail, Miguel A. Bedoya-Pérez, Rhys Cohen, Vicki Kotsirilos, Iain S. McGregor, Elizabeth A. Cairns

**Affiliations:** ^1^ The Lambert Initiative for Cannabinoid Therapeutics, The University of Sydney, Sydney, NSW, Australia; ^2^ School of Psychology, The University of Sydney, Sydney, NSW, Australia; ^3^ School of Social Sciences, Department of Sociology, Macquarie University, Sydney, NSW, Australia; ^4^ Cannabis Consulting Australia, Sydney, NSW, Australia; ^5^ NICM Health Research Institute, Western Sydney University, Sydney, NSW, Australia

**Keywords:** medicinal cannabis, prescribing trends, Australia, authorised prescriber scheme, special access scheme, cannabinoid, regulation, therapeutic goods administration (TGA)

## Abstract

A regulatory framework allowing legal access to medicinal cannabis (MC) products has operated in Australia since November 2016. MC prescribing by healthcare practitioners (HCPs) is primarily conducted through the Special Access Scheme - Category B (SAS-B) pathway, through which prescribers apply to the Therapeutic Goods Administration (TGA–the federal regulator) for approval to prescribe a category of product to an individual patient suffering from a specific indication. The dataset collected by the TGA provides a unique opportunity to examine MC prescribing trends over time in the Australian population. Here we analysed this TGA SAS-B dataset since inception with respect to age, gender, product type (e.g., oil, flower, etc.), CBD content, indication treated, and prescriber location. Results are presented descriptively as well as being analysed using non-linear regression models. Relationship between variables were explored via correspondence analyses. Indications were classified with reference to the International Statistical Classification of Diseases and Related Health Problems (10th Revision). As of 31 August 2021, a total of 159,665 SAS-B approvals had been issued for MC products, 82.4% of were since January 2020. Leading indications for approvals were for pain, anxiety, and sleep disorders. Oil products were the most popular product type, while CBD-dominant products (≥98% CBD) accounted for 25.1% of total approvals. Approvals for flower products increased markedly during 2020–2021, as did approvals involving younger age groups (18–31 years old), male patients, and non-CBD dominant products. A disproportionate number of SAS-B MC applications (around 50%) came from HCPs in the state of Queensland. Associations between patient gender and age and/or indication with product type were found. For example, approvals for oil products were commonly associated with approvals for pain. While, overall prescribing increased dramatically over the last 2 years of analysis, stabilization of approval numbers is evident for some indications, such as pain. Current prescribing practices do not always reflect provided TGA guidance documents for MC prescribing. While acknowledging some limitations around the SAS-B dataset, it provides a unique and valuable resource with which to better understand current prescribing practices and utilisation of MC products within Australia.

## 1 Introduction

The use of cannabis as a medicine can be traced as far back as 2000 BCE in Central Asia, where it has documented use in treating a significant array of health problems (Crocq, 2020). The recent worldwide renaissance in the use of cannabis for medical purposes is supported by evidence of efficacy, albeit somewhat variable, across a range of conditions such as chronic pain (Stella et al., 2021), muscle spasticity in multiple sclerosis (Fragoso et al., 2020), chemotherapy-induced nausea and vomiting (Grimison et al., 2020), palliative care (Herbert and Hardy, 2021), and severe forms of childhood epilepsy (Nabbout and Thiele, 2020).

The cannabis plant contains hundreds of bioactive molecules, of which two plant-derived cannabinoids (phytocannabinoids) are the best studied: Δ^9^-tetrahydrocannabinol (THC), the main intoxicating constituent; and cannabidiol (CBD), which is non-intoxicating. THC and CBD have distinct pharmacological actions and different, but partly overlapping, therapeutic applications. THC influences pain, spasticity, sedation, appetite, and mood in animal and human studies, primarily through agonist action on cannabinoid receptors 1 (CB1) and 2 (CB2) (Banister et al., 2019). CBD is more “promiscuous”, having activity at a large number of targets, and with anxiolytic, anti-convulsant and anti-inflammatory effects reported, in at least preclinical models (Nelson et al., 2020).

Evidence around efficacy of cannabis in certain health conditions continues to evolve, with rapidly increasing global numbers of randomized controlled trials (RCTs) and preclinical research (Schlag et al., 2021). However, there remain many conditions for which clinical evidence is minimal or ambiguous, with systematic reviews often highlighting a paucity of high quality randomized RCTs to support current prescribing (Alexander, 2020).

Legal availability of medicinal cannabis (MC) varies by location, and even within the same country there can be differential regulation at state and federal levels (reviewed in Gleeson, 2020). Recent reviews of MC programs can be found elsewhere (Decorte et al., 2020; Corva and Meisel, 2021). Prescribing MC is a relatively new phenomenon in Australia, with the government legalizing a framework for MC access in November 2016 (Gleeson, 2020). Patient access pathways exist under the regulatory power of the *Therapeutic Goods Administration (TGA),* within the federal Department of Health. Almost all currently available MC products are classified as “unregistered medicines” as they have not undergone the rigorous assessment of safety, quality, and efficacy that would allow entry into the *Australian Registry of Therapeutic Goods (ARTG).* The only two current exceptions are *Sativex* (nabiximols, an oromucosal spray containing equivalent amounts of THC and CBD) and *Epidyolex* (also known as *Epidiolex* in other jurisdictions, a 100 mg/ml CBD solution; Therapeutic Goods Administration, 2020a)*,* although other products, such as *Marinol* (THC capsules) and *Cesamet* (a THC analogue called nabilone) are also approved in other countries.

There are three routes through which a healthcare practitioner^1^ (HCP) can request permission from the TGA to prescribe an unregistered MC product to a patient. The *Special Access Scheme Category A* (*SAS-A*) allows practitioners to prescribe MC products to a patient that is seriously ill or likely to die, with only post-hoc notification of prescribing to the TGA being required. Most patient access, however, is through the *Special Access Scheme Category B* (*SAS-B*) which allows practitioners to make an application to the TGA to allow an individual patient to be prescribed a certain type of MC product to treat a specific condition (Therapeutic Goods Administration, 2020b). Prior to November 2021, SAS-B applications were required to nominate a specific product to be prescribed, and so if a patient required more than one product, then multiple applications were needed (Gleeson, 2020). A refinement to the scheme in November 2021 allowed prescribers to request a general class of product, based on cannabinoid content and product format, rather than an individual product. The third and final route is *The Authorised Prescriber* (*AP*) scheme, which allows HCPs an authority to prescribe a specific MC product to multiple patients in their care if the patients all suffer from the same condition.

Although not registered medicines, the TGA do regulate supply and access to MC products to ensure appropriate practices in manufacturing. The one exception to this is compounded medicines, which have been largely exempt from therapeutic goods regulations (Falconer and Steadman, 2017) and currently do not require prescribers to seek TGA approval or post-hoc notification (Therapeutic Goods Administration, 2020c), as described above. However, the government has announced reforms to the regulation of compounded products that are to commence in April 2022 (Therapeutic Goods Administration, 2022).

Medical conditions allowed for MC prescribing are not specified by the TGA although a series of guidance documents were published in 2017 by the TGA, outlining the evidence-base for use of MC products in multiple sclerosis, palliative care, epilepsy (pediatric and young adult patients), nausea and vomiting, and chronic non-cancer pain (Therapeutic Goods Administration, 2017). In general, however, any practitioner can apply to the TGA to prescribe a MC product to a patient for any condition, provided they can justify prescribing based on the available evidence (Benson and Cohen, 2019).

Since the inception of MC access in Australia in November 2016, the TGA has collected detailed information on approvals issued under the SAS-A, SAS-B, and AP schemes. These datasets are accessible and provide a unique repository of detailed patient-level information around MC approvals that covers almost the entire population of Australian MC patients. It is the largest known dataset of its kind with few other countries/regions systematically capturing approvals in this way (see Banerjee et al., 2022).

To date, little systematic analysis of these TGA datasets have been undertaken (although see Benson and Cohen, 2019; Arnold et al., 2020; Henderson et al., 2021). The purpose of the current study was to provide more detailed analysis of these datasets to allow insights into current trends in MC prescribing in Australia, including indications, patient demographics, and product categories. Our analysis of trends stretches back to when a framework for MC access became legally available in Australia (November 2016). Elucidating trends over time may yield novel and valuable insights into current clinical practice and contribute to a more comprehensive understanding of patterns of prescribing.

## 2 Materials and Methods

### 2.1 Data Acquisition

An email request was made to the TGA under *The Freedom of Information Act (1982)* *(FOI)* for the release of documents pertaining to applications through the SAS-B and AP schemes since inception (2016; Supplementary Figure S1). The scope of this request was informed by previously released FOI datasets (FOI 2013, 2250, 2275, 2370, 2419). The data received via FOI request were supplemented (where specified) with data from the new TGA Medicinal Cannabis Access Data Dashboard, which contains publicly available SAS-A and SAS-B data summaries (Therapeutic Goods Administration, 2021a). No alternative source of AP data is currently publicly available. Human ethics approval was not required for this study as it involved data already collected.

### 2.2 Data Preparation

FOI data were systematically ordered on Microsoft Excel (version 16.54). Three applications were excluded from temporal analysis due to application dates being listed as prior to 2016. Age groups were determined by calculating septiles based on frequencies of approvals for ages 18 and older. MC products were grouped into 9 formats (capsules, crystal, flower, lozenge, oil, spray, tablet, topical, and wafer) based on those reported by Freshleaf Analytics (2021). Indications were classified with reference to the International Statistical Classification of Diseases and Related Health Problems 10th Revision (ICD-10; WHO, version 2019—English) to improve consistency and validity of indication analysis. These classifications were then verified for appropriateness by a Medical Practitioner with significant clinical experience and expertise in this area. Where provided indications were ambiguous, or indications were multifactorial, the nearest possible indication classification was chosen.

Applications and patient numbers reported per consulting location were also normalized according to the Australian Bureau of Statistics population statistics in March 2021 (Australian Bureau of Statistics, 2021), reported here as approvals per 100,000 people.

### 2.3 Statistical Analysis

Data were considered at a general descriptive level (e.g., overall numbers of approvals over time by patients, products, indications), as well as being analyzed using best fit non-linear regression models. Statistical analyses were done in R version 4.1.1 (R Core Team, 2021). Data was processed for each analyses using the packages “tidyverse” (Wickham et al., 2019), “padr” (Thoen, 2020) and “dplyr” (Wickham et al., 2021). Examinations of changes over time were performed by non-linear regression fitting using the g*lm* and *glm.nb* functions from the package “MASS” (Venables and Ripley, 2002) and graphs were constructed using the packages “ggplot” (Wickham, 2016), “cowplot” (Wilke, 2020) and “ggpubr” (Kassambara, 2020). Residuals plots and Pearson’s dispersion test were used to choose the appropriate error distribution for each regression fit—i.e., Poisson or Negative binomial. A stepwise comparison of non-linear polynomial regression of the 1st, 2nd, 3rd, and 4th degree was carried out comparing the Corrected Akaike Information Criterion (AICc) for each regression using the package “MuMIn” (Bartoń, 2020) to determine the curve with comparatively best fit, without overfitting the data. Raising the functions to a higher degree allowed the model to fit more turning points or fluctuations in the data, indicating increasing complexity in the pattern of change. Here, Δm was calculated between models and excluded models with Δm > 2 as having substantially less support (Burnham and Anderson, 2002). To estimate goodness of fit, R^2^ was calculated for each of the best fitted regressions by the equation: 1—deviance/residual deviance, and using the classification established by Moore and Kirkland (2013). Averages are listed as means ± standard error unless otherwise specified.

Correspondence analyses were performed using packages “Factoshiny” and “FactoMineR” (Le et al., 2008; Vaissie et al., 2021). These display associations between two categorical variables, taking into account weighting according to frequency. A graphical representation of the relationship between categorical variables is constructed by plotting the two most explanatory dimensions based on residuals. These dimensions explain the proportion of variance that is displayed along a horizontal or vertical axis, the sum of which explains the total variance represented in the graph. The point at which the two-axis intercept (origin) represents the point of least differentiation, and those categories close to this point can be considered to deviate the least from expected proportions.

Three separate insights into the properties of the two categorical variables can be interpreted from this analysis. First, the categories further away from the origin are considered more differentiated. Second, the proximity of two categories from the same variable, for example two different product formats, indicates that they are probably similar. Finally, a greater association is demonstrated by a smaller angle between the vectors connecting two categorical variables to the origin (e.g., between an indication and product).

Graphpad Prism (version 9.2.0) was also used to present the remaining distributions of data across categorical variables.

## 3 Results

On 20 October 2021 the TGA granted the FOI request (FOI 2989), releasing a number of Excel documents by email. The SAS-B dataset contained a chronological record of 159,665 approved applications that were submitted by HCPs between 10 February 2016 and 31 August 2021, and approved between 26 February 2016 and 8 September 2021. Data included indication treated, product format sought, duration of supply, demographic information and whether the patient had received a prior SAS approval for MC. Two applications had been rejected by the TGA and were not included with the dataset (TGA, *personal communication*). Some data points in this set were missing: schedule, 18 applications (0.01%); patient gender, 641 applications (0.40%); state, 52 applications (0.03%); indication, 2 applications (0.00%); product, 18 applications (0.01%); age, 44 applications (0.02%).

In addition to the SAS-B data, AP data were released by the TGA in two additional documents. One document contained the consulting location (state or territory) of Authorised Prescribers and the number of patients either commencing or continuing treatment across five 6-month time periods from June 2016 to December 2020 (*N* = 25,933). The other document listed the indications for which Authorised Prescribers had been approved from May 2018 until September 2021. It included the approval and expiration dates of the authorizations, the number of new patients commenced treatment (*N* = 7,137), and the total number of patients treated over that time (*N* = 10,323).

SAS-A information was not requested via the FOI but is available on the Medicinal Cannabis Access Data Dashboard. As of 23 March 2022, 1,398 SAS-A notifications have been logged by the TGA (Therapeutic Goods Administration, 2021a).

### 3.1 Approved SAS-B Applications Over Time

**FIGURE 1:**
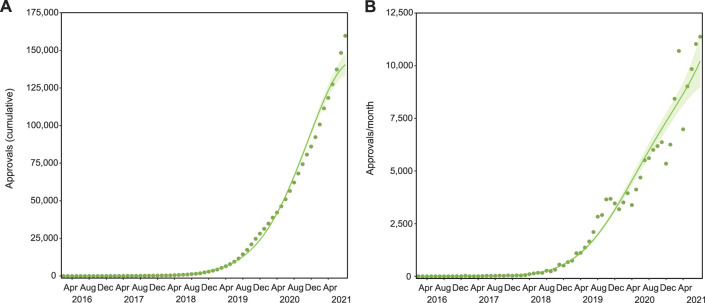
SAS-B approvals over time. There have been 159,665 cumulative SAS-B approvals since 2016, with growth that followed a Negative binomial, 3rd degree polynomial curve (R^2^ = 0.998, Δm = 44.398; **(A)**. Applications per month increase by around 12,000 a month, following a Negative binomial, 4th degree polynomial curve (R^2^ = 0.984, Δm = 22.590; **(B)**. Solid lines represent the best fit with shaded standard error of the mean (SEM).

The number of SAS-B approvals has been increasing in a non-linear pattern over time (3rd degree polynomial, *R* ^*2*^ *=* 0.998, Δm = 44.398) with a dramatic increase in the last 2 years of analysis. Indeed, only 1.80% of total cumulative approvals were granted in the first 3 years of the legal access (2016–2018; Figure 1A). The rate of growth throughout the entire period (2016–2021) was non-linear (Figure 1B; 4th degree polynomial, *R* ^*2*^ = 0.984, Δm = 22.590), and does not yet appear to be slowing. For example, applications submitted between January and August 2021 were 2.2 times greater compared to the equivalent period in 2020.

### 3.2 Indications

There were 202 unique entries for indications specified by practitioners in their SAS-B applications. Reclassifying these according to the ICD-10 found 149 distinct indications, covering 121 different categories within 17 diagnostic groups (Supplementary Table S1). For example, indications listed as “Autism” were reclassified as “childhood autism (F84.0)" as indication, “pervasive developmental disorders (F84)" as category, which falls under the “mental and behavioral disorders (V)" diagnostic group. An additional 5 indications were insufficiently described to be coded (see “inadequate information”, Supplementary Table S1).

**FIGURE 2:**
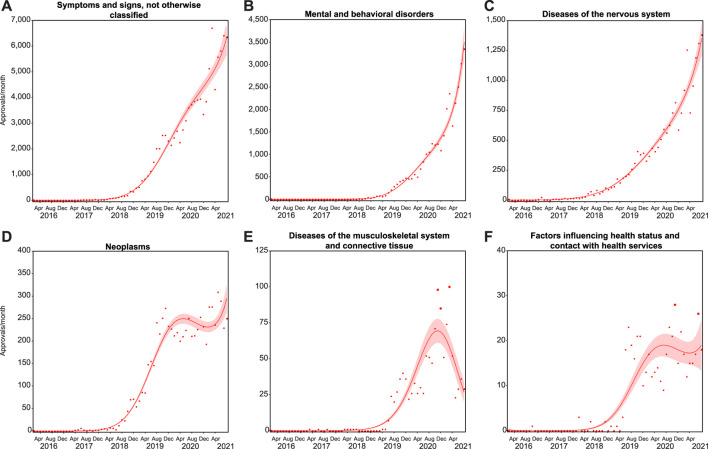
Growth across indication groups differs. Trends in monthly approvals over time were analyzed in six ICD-10 groups representing almost all cumulative approvals (Supplementary Table S1). Approvals for symptoms, signs and abnormal clinical and laboratory findings, not elsewhere classified **(A)**; mental and behavioral disorders **(B)**; neoplasms **(C)**; and diseases of the nervous system **(D)** followed Negative binomial, 4th degree polynomial curves (R^2^ = 0.989, 0.993, 0.976, and 0.976, and Δm = 7.055, 18.903, 20.462, and 6.037, respectively). Approvals for diseases of the musculoskeletal system and connective tissue **(E)**; and factors influencing health status and contact with health services **(F)** followed Negative binomial 3rd degree polynomial curves (R^2^ = 0.894 and 0.831, and Δm = 83.058 and 49.676, respectively). Solid lines represent the best fit with shaded standard error of the mean.

Almost all indications fell into 6 diagnostic groups: “symptoms, signs and abnormal clinical and laboratory findings, not elsewhere classified” (symptoms and signs; *N* = 100,744); “mental and behavioral disorders” (*N* = 31,315); “diseases of the nervous system” (*N* = 18,463); “neoplasms” (*N* = 6,984); “diseases of the musculoskeletal system and connective tissue” (musculoskeletal; *N* = 1,616); and “factors influencing health status and contact with health services” (health services; *N* = 476; Figure 2). While the number of monthly applications for indications falling in these first three groups continues to rise (symptoms and signs, 4th degree polynomial, *R* ^*2*^ = 0.989, Δm = 7.055; mental and behavioral disorders, 4th degree polynomial, *R* ^*2*^ = 0.993, Δm = 18.903; diseases of the nervous system, 4th degree polynomial, *R* ^*2*^ = 0.976, Δm = 6.037), the change in the number of monthly applications for the next three groups appears to be more stochastic (neoplasms, 4th degree polynomial, *R* ^*2*^ = 0.976, Δm = 20.462; musculoskeletal, 3rd degree polynomial, *R* ^*2*^ = 0.894, Δm = 83.058; health services, 3rd degree polynomial, *R* ^*2*^ = 0.831, Δm = 49.676).

**FIGURE 3:**
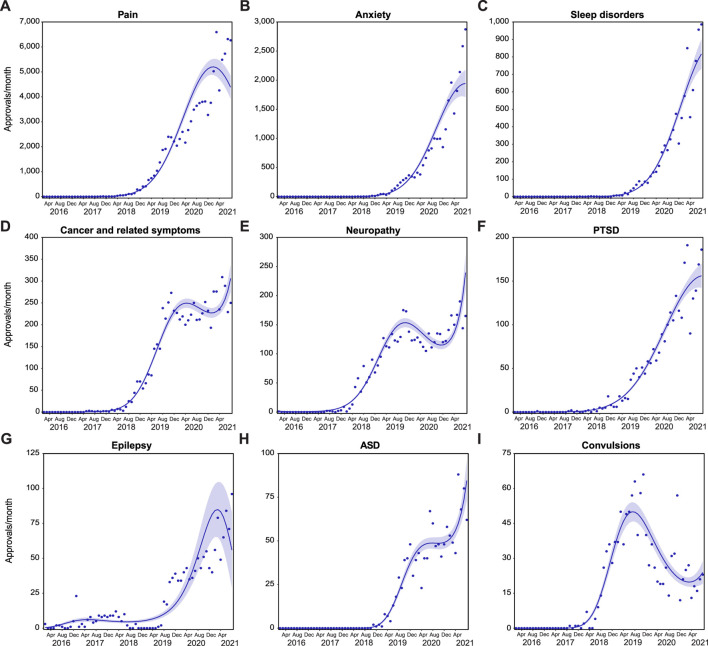
Growth is varied across indication categories. Trends in monthly approvals over time were analyzed in ICD-10 indication categories with >1,000 cumulative approvals (Supplementary Table S1). Approvals for pain **(A)**; anxiety **(B)**; sleep disorders **(C)**; and PTSD **(F)** followed Negative binomial, 2nd degree polynomial curves (R^2^ = 0.988, 0.989, 0.987, and 0.973, and Δm = 13,202.020, 1,413.481, 51.913, and 54.544, respectively). Approvals for cancer and related symptoms **(D)**; and neuropathy **(E)** followed Negative binomial 4th degree polynomial curve (R^2^ = 0.981 and 0.962, and Δm = 17.271 and 21.698, respectively). Approvals for epilepsy **(G)** moderately followed a Negative binomial 4th degree polynomial curve (R2 = 0.587, Δm = 7.922). Approvals for ASD **(H)** and convulsions **(I)** followed Negative binomial 3rd degree polynomial curves (R^2^ = 0.977 and 0.918, and Δm = 27.715 and 31.812, respectively). Solid lines represent the best fit with shaded standard error of the mean.

Nine ICD-10 indication categories had over 1,000 cumulative approvals in the SAS-B dataset (Figure 3), representing 94.1% of total approvals. These conditions were “pain, not elsewhere classified” (61.0% of total approvals); “other anxiety disorders” (16.0%); “sleep disorders” (5.7%); “neoplasm of uncertain or unknown behavior of other and unspecified sites” (4.4%); “other polyneuropathies” (3.0%); “reaction to severe stress, and adjustment disorders” (1.6%); “epilepsy” (0.8%); “pervasive developmental disorders” (0.8%); and “convulsions, not elsewhere classified” (0.8%). These are referred to henceforth as pain; anxiety; sleep disorders; cancer and related symptoms; neuropathy; PTSD; epilepsy; ASD; and convulsions, respectively, for the remainder of the text.

The majority of these indication categories show continued growth in SAS-B approvals over time, with the exception of an apparent slowing of approvals for pain (Figure 3A; 2nd degree polynomial; *R* ^*2*^ = 0.988, Δm = 13,202.020); a trend towards decreasing monthly approvals in epilepsy (Figure 3G; 4th degree polynomial, *R* ^*2*^ = 0.587, Δm = 7.922); and an increase following a prior decrease in the number of monthly approvals for convulsions (Figure 3I; 3rd degree polynomial, *R* ^*2*^ = 0.918, Δm = 31.812).

### 3.3 Products

There are currently at least 375 different unregistered MC products in Australia that can be supplied via the SAS-B and AP schemes (Therapeutic Goods Administration, 2021b; Freshleaf Analytics, 2021). These include different formulations and composition (e.g., capsules, sprays, oils, flower). Different routes of administration may be optimal for different conditions (Bruni et al., 2018). For example, inhaled routes of administration have rapid absorption, and onset of action within seconds to a few minutes which may be useful for breakthrough pain (Malcolm, 2018). On the other hand, oral products such as oils and capsules have much slower onset to action, and more persistent effects (Vandrey et al., 2017).

In Australian regulation, the THC and CBD content of MC products determines the “Schedule” they fall under in the “Poisons Standards”, based on the potential risks and harms associated with their use (Therapeutic Goods Administration, 2020b). Products comprising ≥98% CBD in total cannabinoid content are in Schedule 4 (*Prescription Only Medicine*) reflecting an acceptable safety profile (Iffland and Grotenhermen, 2017; World Health Organization, 2018). By contrast, THC has intoxicating properties and has abuse potential (Banister et al., 2019). During the project period, MC products that contain <98% CBD, and therefore likely higher THC content, were classified as Schedule 8 (*Controlled Drug*) in most states in Australia. In addition to federal approval by the TGA, prescribers may also need approval from their state or territory health department, although the conditions under which state/territory approval is required varies considerably between jurisdictions.

**FIGURE 4:**
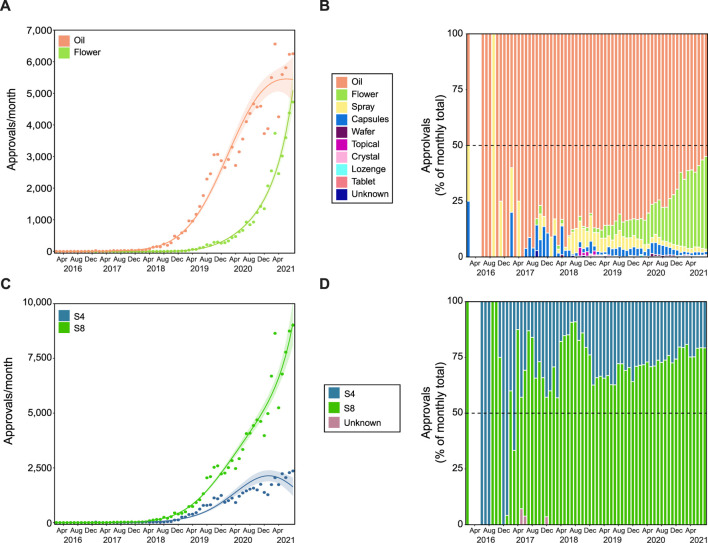
Flower and Schedule 8 products have disproportionate growth. Growth trends in product format **(A,B)** and product schedule **(C,D)**. Approvals for oil products followed a Negative binomial 4th degree polynomial curve (R^2^ = 0.980, Δm = 18.292), while approvals for flower followed a Negative binomial 3rd degree polynomial curve (R^2^ = 0.991, Δm = 8.267). The proportion (%) of approvals per product category (see legend) are shown in panel **(B)**. Approvals for S4 and S8 products followed Negative binomial 4th degree polynomial curves (C; R^2^ = 0.934 and 0.987, and Δm = 11.691 and 15.707, respectively). The proportion (%) of approvals per product schedule (see legend) are presented in panel **(D)**. The gap (between February 2016 and June 2016 in panels **(B)** and **(D)**, indicates no applications submitted in this period. In panels **(A)** and **(C)** Solid lines represent best fit with shaded standard error of the mean.

The FOI data received did not include information on individual products, only the Schedule (i.e., Schedule 4 [S4] or 8 [S8]) and product format (e.g., oil, flower, capsule). There were 45 variations of product format that were specified in approved applications to the TGA, with oil and flower products representing >90.0% of total cumulative approvals (Supplementary Table S2; Figure 4). On average, 79.8 ± 2.1% of applications each month were for oil products, while 9.1 ± 1.5% were for flower (Figure 4B). The number of applications for oil increased over time (Figure 4A; 4th degree polynomial, *R* ^*2*^ = 0.980, Δm = 18.292), while applications for flower products showed a more rapid increase from the end of 2019 (3rd order polynomial, *R* ^*2*^ = 0.991, Δm = 8.267).

Overall, the majority of approvals were for S8 products (74.8%), with a pronounced non-linear growth (4th degree polynomial, *R* ^*2*^ = 0.987, Δm = 15.707; Figures 4C,D), while growth in S4 approvals appears to be slowing (4th degree polynomial, *R* ^*2*^ = 0.934, Δm = 11.691). This is consistent with trends seen with the top nine indications (Supplementary Figure S2). Approvals for S8 products gradually increased over time for anxiety conditions (1st degree polynomial, *R* ^*2*^ = 0.742; Supplementary Figure S2B). Until early 2019, approvals for anxiety conditions were primarily for S4 products, but by August 2021, 78.2% were for S8 products. Additionally, while S4 approvals were more common overall in epilepsy (Supplementary Figure S2G), ASD (Supplementary Figure S2H) and convulsions (Supplementary Figure S2I), current prescribing patterns appear to be trending toward majority S8 approvals in epilepsy (3^rd^ degree polynomial, *R* ^*2*^ = 0.656, Δm = 4.482) and convulsions (3^rd^ degree polynomial, *R* ^*2*^ = 0.375, Δm = 7.864, weak fit). The remaining indications were either relatively stable or stochastic.

### 3.4 Patient Demographics

**FIGURE 5:**
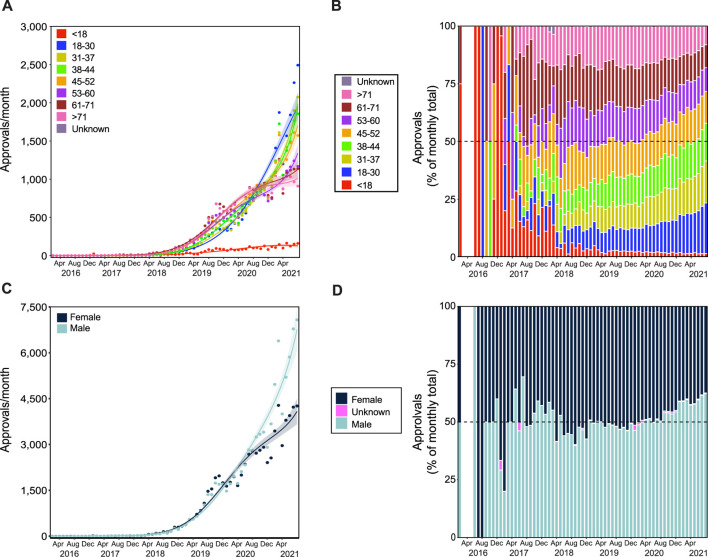
Patient demographics for SAS-B approvals. Growth trends in age groups **(A,B)** and in patient genders **(C,D)**. Approvals for patients younger than 18 years and between 31 and 37 years of age followed a Negative binomial 3rd degree polynomial curve (R^2^ = 0.888 and 0.993, and Δm = 6.750 and 22.056, respectively); approvals for patients between 18 and 30 years of age followed a Negative binomial 2nd degree polynomial curve (R^2^ = 0.986, and Δm = 58.345); approvals for patients in age groups 38 to 44, 45 to 52, 53 to 60, 61 to 71, and over 71 years of age followed a Negative binomial 4th degree polynomial curve (R^2^ = 0.990, 0.990, 0.991, 0.985, and 0.989, and Δm = 10.929, 16.765, 10.162, 27.937, and 8.374, respectively). Panel **(B)** shows the proportion of approvals for each age category (see legend) per month. Approvals for females and males followed Negative binomial 4th degree polynomial curves (**C**; R^2^ = 0.986 and 0.989, and Δm = 32.522 and 16.797, respectively). The proportion (%) of approvals by patient gender per month are shown in panel **(D)**. Solid lines in panels **(A)** and **(C)** represent best fit with shaded standard error of the mean. The gap (between February 2016 and June 2016 in panels **(B)** and **(D)**, indicates no applications submitted in this period.

Patient ages ranged from 0–103, which were grouped into the following categories: <18 (*N* = 2,978); 18–30 (*N* = 23,635); 31–37 (*N* = 21,968); 38–44 (*N* = 22,667); 45–52 (*N* = 23,390); 53–60 (*N* = 20,848); 61–71 (*N* = 22,599); >71 (*N* = 21,537), and unknown (*N* = 43). Prior to 2019, the majority of approvals were granted for patients >45 years old (Figures 5A,B). However, since 2019, the proportion of approvals for younger patients is increasing, with the exception of patients <18 (3rd degree polynomial, *R* ^*2*^ = 0.888, Δm = 6.750). Approvals for patients aged 18–30 have been steadily increasing (2nd degree polynomial, *R* ^*2*^ = 0.986, Δm = 58.345), and comprised the largest number of approvals for 2021 (18.6%), particularly in August 2021, where they represented 21.9% of all approvals. Approvals for patients between 31 and 37 are increasing rapidly (3rd degree polynomial, *R* ^*2*^ = 0.993, Δm = 22.056), making up 16.1% of all approvals in 2021. These proportional gains are likely achieved from the relative lack of growth in the number of applications for patients aged 61–71 (4th degree polynomial, *R* ^*2*^ = 0.985, Δm = 27.937), and >71 (4th degree polynomial, *R* ^*2*^ = 0.989, Δm = 8.374).

Since early 2020, the rate of increase in approvals has also been greater in males compared with females (Figure 5C; 4th degree polynomial, *R* ^*2*^ = 0.989, Δm = 16.797, and 4th degree polynomial, *R* ^*2*^ = 0.986, Δm = 32.522, respectively), which is reflected in the observed proportional gains. In January 2020, 46.3% of approvals were for males, but in August 2021 was 62.2% (Figure 5D).

### 3.5 Prescriber Consulting Location

**FIGURE 6:**
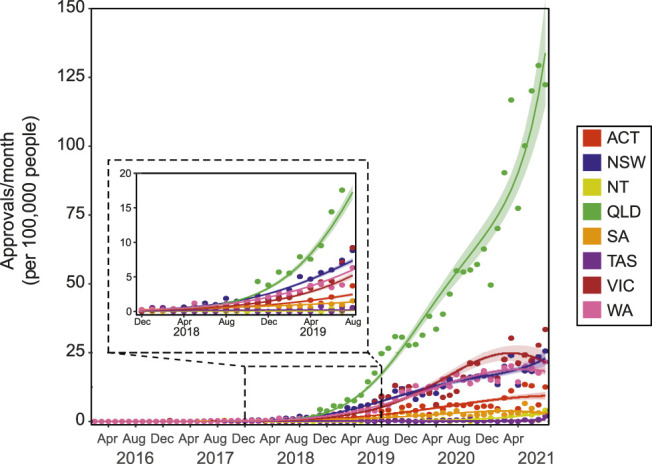
SAS-B approvals across states and territories. The number of SAS-B approvals per month across states and territories represented as per 100,000 persons. Approvals from the Northern Territory (NT) followed a Poisson 1st degree polynomial curve (R^2^ = 0.772). Approvals from Australian Capital Territory (ACT) and Western Australia (WA) followed Negative binomial, 2nd degree polynomial curves (R^2^ = 0.926 and 0.986, and Δm = 24.998, and 1,525.398, respectively). Approvals Victoria (VIC) followed a Negative binomial, 3rd degree polynomial curves (R^2^ = 0.934, Δm = 11.759). Approvals New South Wales (NSW), Queensland (QLD) and South Australia (SA) followed Negative binomial, 4th degree polynomial curves (R^2^ = 0.982, 0.986, and 0.926, and Δm = 6.783, 73.955, and 4.143, respectively). Approvals from Tasmania (TAS) moderately followed a Negative binomial, 3rd degree polynomial curve (R^2^ = 0.402, Δm = 2.611). Solid lines represent best fit with shaded standard error of the mean.

The SAS-B prescribing trends also varied by prescriber consulting location. Australia has 6 states and 2 territories, which, as described above, can sometimes differ in how scheduled drugs are regulated. Prior to 2019, the rate of prescribing normalized to population (per 100,000) was relatively comparable between states. However, the normalized rate of growth of approvals from the state of Queensland far outnumbers all other states and territories (Figure 6; 4th degree polynomial, *R* ^*2*^ = 0.986, Δm = 73.955). A trend toward continued growth was observed in the remaining states and territories, with the exception of Victoria (3^rd^ degree polynomial, *R* ^*2*^ = 0.934, Δm = 11.759).

### 3.6 Authorised Prescribers

Authorised Prescribers are required to report to the TGA twice a year on the number of patients they have treated in the prior 6 months. AP data were provided by the TGA in two documents that were grouped differently, either by indication, or by state. They also contained overlapping, but non-matching time frames. Further, when selecting for approximately the same time frame (2018–2020), these documents contained different reporting numbers. When sorting by indication, there were 6,748 and 9,687 reports for new and continuing patients, respectively. When sorting by location, there were 15,333 new patient reports, and 10,210 continuing. When asked to clarify the discrepancies between these two datasets, a TGA spokesperson commented that “this is incomplete data and it is the best we could provide with what information is available to us. They are not linked” (TGA, *personal communication*). In general, pain was the most common indication category, and most approvals originated from prescribers in Queensland. Given the unreliability and low quality of these data, and the lack of patient demographics and product information, a more meaningful analysis of this dataset could not be completed. However, monitoring of information released by the TGA over time indicates that the number of registered APs has been increasing significantly in the last year, with 194 active APs in January 2021, rising to 715 in January 2022 (data not shown).

### 3.7 Associations

**FIGURE 7:**
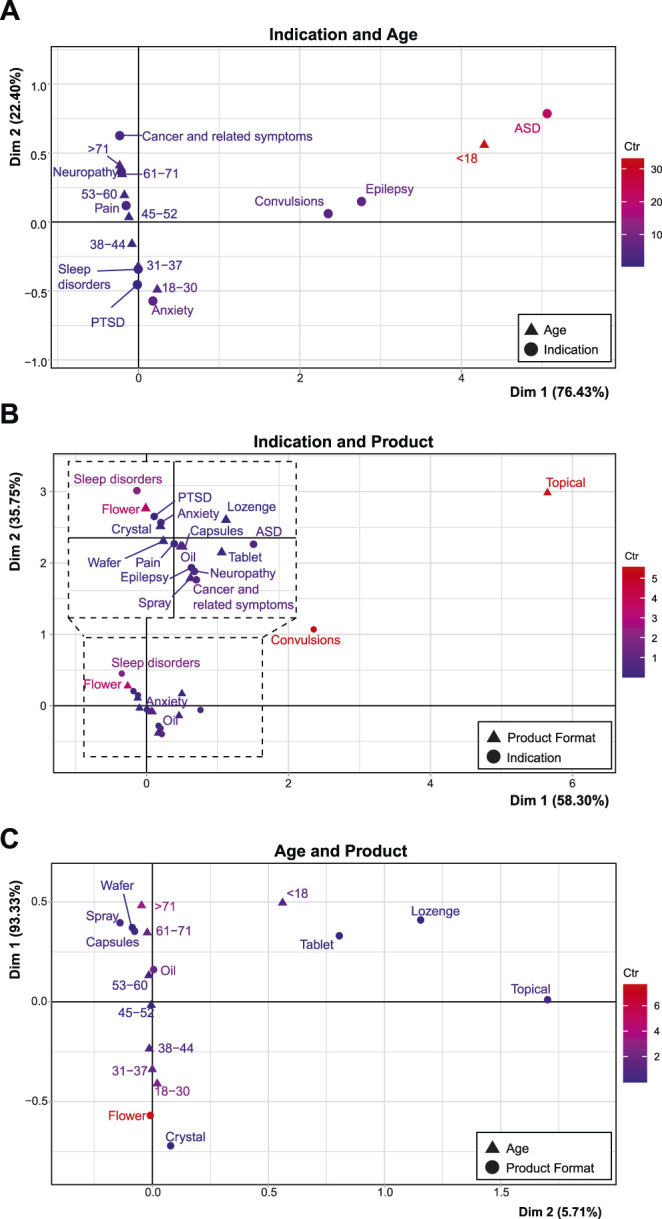
Associations between age, product format, and indication. Correspondence analyses with groups of age compared with indication **(A)**, indication compared with product preference (with an inset representing an expanded view of the dashed area; **(B)**, and age compared with product preference **(C)**. Description of deviation from independence is labelled with the according axes, while the red to blue color gradient indicates the scaled contribution of these factors to the overall variance (the inertia*100; “Ctr”) for each graph. The maximum inertia*100 are 33.07, 8.47, and 5.56, respectively. See Supplementary Tables S3–S5 for the contribution of variance related to these graphs.

To probe the relationship between key variables, a two-dimensional correspondence analysis was used. This analysis captured distinct patient subgroups with attributes of indication category, product category, and age group. Associations were found between indication category and patient age group (χ^2^_56_ = 69,422.45, *p* < 0.001; Figure 7A), indication and product format (χ^2^_64_ = 16,143.57, *p* < 0.001; Figure 7B), and product format and age group (χ^2^_56_ = 69,490.8, *p* < 0.001; Figure 7C). In each instance, variables with a larger contribution than the expected average were considered distinct subgroups. Cut off for age was 12.5%, and for indication and product type was 11.1%. A summary of the contribution to variance in each of these studies is included in Supplementary Tables S3–S5.

In comparing indication and age, patients aged <18 deviated from the average with a large contribution to overall variance (Dim 1: 92.01%, inertia*100 = 33.12) and were largely associated with ASD (Dim 1: 60.91%, inertia*100: 22.06), as well as epilepsy (Dim 1: 19.38%, inertia*100 = 6.87), and convulsions (Dim 1: 12.77%, inertia*100 = 4.52). There were also associations along the Dim 2 axis, between patients aged 18–30 (Dim 2: 34.88%, inertia*100 = 4.53) and approvals for anxiety (Dim 2: 54.0%, inertia*100 = 6.22). Another subgroup were patients aged >71, who were more associated with approvals for neuropathy and cancer and related symptoms (Dim 2: 21.11%, inertia*100 = 2.98), as well as patients aged 61–71 (Dim 2: 16.04%, inertia*100 = 2.37). Each age category was associated with a particular indication with no average profile observed.

Approvals for topical products contributed greatly to the Dim 1 axis construction (68.53%, inertia*100 = 5.56) when investigating product vs. indication. This group was associated with approvals for convulsions (Dim 1: 70.93%, inertia*100 = 5.45). Approvals for flower products also represented a distinct patient subgroup (Dim 2: 47.15% inertia*100 = 3.44), that was significantly related to sleep disorders (Dim 2: 32.11%, inertia*100 = 2.03). In this analysis, pain represented the average profile of application across all products (inertia*100 = 0.2, coordinate for Dim 1: 0.00 and Dim 2: 0.04).

Approvals for flower products were the most distinct patient subgroup in relation to age group, as indicated by the distance from the origin and the highest relative contribution to inertia (contribution to Dim 1: 73.80%, inertia*100 = 7.66). This was associated with patients aged 18–30 (contribution to variance Dim 1: 23.70%, inertia*100 = 2.48) and to a lesser extent 31–37 (contribution to variance Dim 1: 15.01%, inertia*100 = 1.56). Approvals for oil products also contributed to variance (contribution to variance Dim 1: 17.58%, inertia*100 = 1.84), and was associated with patients aged 53–60. Sprays, wafers, and capsules were associated with applications for patients aged 61–71 (contribution to variance Dim 1: 16.58%, inertia*100 = 1.74), and >71 who were a distinct patient subgroup (contribution to variance Dim 1: 30.57%, inertia*100 = 3.24). Patients aged 45–52 represented the average profile, having little contribution to the overall variance and being in close proximity to the origin (inertia*100 = 0.01, coordinate for Dim 1: 0.02 and Dim 2: 0.05), with no association to a specific product choice.

## 4 Discussion

The current report characterizes the prescribing of MC products under the SAS-B scheme in Australia since the inception of a legal MC framework in November 2016. The availability of a unique large dataset with detailed patient-level data provides an unparalleled opportunity to examine MC prescribing trends in Australia. This analysis represents the first step in what could possibly be a series of future analyses, including expanded analysis within particular subsets of data, incorporating additional information as it becomes available, as described below.

### 4.1 Trends Over Time

The SAS-B dataset shows dynamic and evolving prescribing trends, not necessarily foreseeable at the inception of the scheme in 2016. The dramatic escalation of prescribing over time is unlikely to reflect greater population morbidity (with notable exceptions, *vide infra*), but is more likely to reflect improved patient access pathways and greater familiarity and acceptance of MC prescribing amongst HCPs. Surveys of Australian health professionals report a shifting attitude towards acceptance of MC as a treatment option, as more educational material and evidence becomes available, and prescribers become more confident in their MC prescribing practices (Karanges et al., 2018; Lewis and Flood, 2021). Also salient was the launch of a streamlined online “portal” system for SAS-B applications in 2018, with the intention of improving the speed and simplicity with which clinicians could apply for SAS-B MC approvals (Therapeutic Goods Administration, 2020b).

Policy changes since 2016 are also relevant, as some state and territory-level eligibility and approval requirements have been simplified or removed (Royal Australian College of General Practitioners, 2019). For example, in 2017, Queensland restricted general practitioners from prescribing without the endorsement of a condition-specialist physician, and required state Health Department approval (in addition to TGA approval) for all MC prescriptions (Public Health (Medicinal Cannabis) Act 2016 (Queensland)). Reforms made in July 2019 allowed all HCPs to prescribe, and the requirement for state approval for MC prescribing was reduced to S8 products and only when prescribing to drug-dependent patients (Health Legislation Amendment Regulation (No.2) 2019 (Queensland)). Similar reforms have been made in other jurisdictions, notably in New South Wales and Victoria (Royal Australian College of General Practitioners, 2019; Community Affairs References Committee, 2020).

Some recent increases in prescribing may be reflective of an overall increase in mental health-related morbidity. An overall increase in mental health-related government-subsidised and co-payment prescriptions has been noted in 2020–2021, likely related to the mental health burden of COVID-19 restrictions and lockdowns in Australia (Australian Institute of Health and Welfare, 2021). MC approvals for mental and behavioural disorders also significantly increased during this period.

In contrast, SAS-B applications for epilepsy showed downward trends. It is possible that this trend may be influenced by the CBD-containing medicine *Epidyolex* becoming a registered medicine in Australia (September 2020), obviating the need for access to CBD under the SAS-B schemes (Therapeutic Goods Administration, 2020b). Interestingly, the proportion of approvals for S8 products for epilepsy has increased since mid-2019, perhaps reflecting inadequate treatment responses in patients with S4 products (Anderson et al., 2020). Future work should compare the changepoints in this (and other) approval data to time-relevant contextual changes (e.g., legislative changes).

Another notable trend is for younger males and females gaining approvals for flower products for indications within the mental and behavioural disorders group, as also noted by Lane and Cohen (2021). This was also evident in correspondence analyses that highlighted this distinct patient subgroup, and a shift toward S8 products in the treatment of anxiety. In some patient scenarios (e.g., breakthrough pain (Bhaskar et al., 2021) and panic attacks (Stith et al., 2020)) vaporized flower is preferred due to the rapid onset of action and shorter duration of action compared to oral products (Huestis, 2007). Future analyses may wish to explore this specific association further.

### 4.2 Disparities Between Prescribing and Provided Therapeutic Goods Administration Guidance

The SAS-B application process requires HCPs to provide a clinical justification for prescription of MC products, drawing on available evidence. The TGA provides *Clinical Guidance* documents to help support this process, but these are limited to indications deemed to have the highest quality evidence (chronic non-cancer pain; epilepsy; palliative care; chemotherapy-induced nausea and vomiting; and spasticity in multiple sclerosis) (Therapeutic Goods Administration, 2017). Notably, there are no guidance documents for leading conditions in the current dataset such as anxiety (Black et al., 2019), sleep disorders (Suraev et al., 2020), ASD (Fletcher et al., 2022), and PTSD (Hindocha et al., 2020). These conditions, all which have >1,000 cumulative approvals, are characterized by ongoing uncertainty around MC efficacy and poor quality of available evidence. Regardless, HCP justification provided in the SAS-B applications was evidentially sufficient to warrant approval by the TGA.

Practitioners may see MC as a viable treatment option, even with limited or ambiguous clinical evidence of efficacy or as last resort treatment when all other conventional treatments have failed (Hallinan et al., 2021). This may be the case with pain and mental and behavioral disorders in particular, where there has been ever-expanding clinical need, and a high side-effect burden with conventional prescribing options (Deloitte Access Economics, 2019; Braund et al., 2021; Painaustralia, 2021).

### 4.3 Identifying Gaps in Clinical Evidence

The SAS-B dataset may be particularly useful in identifying indications where MC treatment effects might not be captured in existing RCT data, either negatively or positively. Association analyses might also assist in identifying subset populations, particularly where approvals are not abundant. For example, we were surprised to find an association between topical products and approvals for convulsions; however, on further examination, we found at least one topical CBD product that is being investigated for use for the treatment of seizures, with this route of administration intended to reduce possible side effects seen with oral administration (O’Brien, 2019; Sebree et al., 2016). Future studies may also assess the utility of a multiple correspondence analysis to allow insights into multiple associations within the SAS-B dataset, rather than the restricted two-dimensional correspondence analysis used in this study.

It is important to note that the SAS-B dataset is essentially descriptive and provides no information around efficacy, or the lack thereof, across indications. It might be presumed that the presence of tens of thousands of prescriptions for an individual condition (e.g., pain) must indicate efficacy, but this is not assured. Repeat applications for the same patient for the same condition might also be considered a proxy for perceived efficacy. However, some conditions may resolve and no longer require treatment, meaning that discontinuation outcomes are inherently ambiguous. Prescribing could also be influenced by the now-available public data on the TGA dashboard (Therapeutic Goods Administration, 2021a), which could create a “feed-forward” cycle in prescribing, even in the absence of perceived patient benefit. Furthermore, while the TGA sets quality standards to ensure consistent cannabinoid content in products used in the SAS and AP schemes (Therapeutic Goods Administration, 2020b), limited information on these products is supplied by product companies to the TGA (Therapeutic Goods Administration, 2021b; Office of Drug Control, 2021). The TGA also does not qualify or verify this information, so the database is likely incomplete and inaccurate. Information on cannabinoid content, dose, and how different conditions have been dosed over time, would also be valuable, especially if coupled to readouts of perceived efficacy.

### 4.4 Caveats and Limitations

Several limitations are important to consider when interpreting this dataset and analysis. While the SAS-B dataset covers the large majority of MC prescribing in Australia, some patient cohorts are not represented, namely, patients receiving products through SAS-A and AP schemes, those receiving compounded products, and those enrolled in clinical trials. To the best of our knowledge, data on patients receiving compounded products or participating in active clinical trials are not collected by the TGA. This changed from March 2022 where the TGA requires prescribers to seek approval via the SAS-B or AP pathways prior to prescribing a compounded MC product. Additionally, while the TGA collects data on the AP scheme, the datasets supplied were incomplete and ambiguous, and so the exact number of patients could not be estimated.

Another significant limitation is the lack of a definitive link between a SAS-B application being approved, a prescription being written, and a product actually being dispensed to a patient (Prof. Nick Lintzeris, *personal communication*). A prescriber may submit multiple SAS-B applications for a single patient but only write a prescription for one product. Alternatively, or in addition, a prescription may be received by a patient but not filled. Until November 2021, the TGA specified that SAS-B and AP applications be for a single named product (Skerritt, 2017; Therapeutic Goods Administration, 2021c), meaning that if a specified product were unavailable from a manufacturer, an additional application needed to be submitted. To overcome this, multiple simultaneous applications for different products were often submitted for the same patient (Prof. Nick Lintzeris, *personal communication*). This has the capacity to distort the SAS-B dataset to over-estimate number of prescriptions. New TGA regulations as of November 2021 allow prescribers to seek an approval to prescribe any products of the same format (e.g., oral oils) in the same product “category” based on cannabinoid content (Therapeutic Goods Administration, 2021c). These changes were implemented to reduce the administrative burden on prescribers who were seeking multiple approvals for the same patient as a hedge against product unavailability. The data collected and used in this paper pre-dates these changes, but future analysis should consider the impact of these changes on MC approvals. SAS-B approvals and AP registration status also have expiration dates, after which they become invalid and must be renewed/replaced. Variations in these timeframes may also impact observed trends.

The data around indication in the current analysis must also be treated with some caution. The SAS-B application process does not require prescribers to specify strict diagnostic criteria, resulting in potentially ambiguous or erroneous classifications of patients. Some applications note patient symptoms rather than diagnosis, for example, some applications noted “epilepsy” (diseases of the nervous system) while others noted “convulsions” (symptoms and signs). It is likely that these approvals involved the same patient population, which, had they been combined, might have produced different trends. Additionally, many conditions are multi-factorial involving multiple medical classifications; for example, MC for “cancer” is ambiguous as to whether the treatment is intended to be targeted at the tumour itself or the *symptoms* associated with cancer and cancer therapy such as nausea, insomnia, or pain. Implementation of a more rigorous diagnostic and data capture process under the current regulatory framework would be a major advantage to future research.

The context in which these data were collected should also be considered, as the regulatory framework and the study of MC continues to evolve. Further analysis of change points in prescribing trends across a broad range of indications within the context of a timeline of published evidence and policy changes would be informative, but is outside the scope of this current work.

Finally, a polynomial regression line of best fit, which assumes that there is infinite potential growth and restricted from negative intercept (i.e., Poisson and Negative binomial error distribution), imposes certain limitations. Using generated trend lines to predict future usage or to model usage in other jurisdictions may be possible, but should be interpreted with caution. A Bass model which incorporates eventual saturation of the available population, would be ideal for appropriately assessing the capacity for growth in the future (Burnham and Anderson, 2010). However, the point of saturation is difficult to estimate given there is no current model at which to estimate this point. Thus, for the purposes of this current analysis, the polynomial model is appropriate. Approvals in some indications, such as pain, seem to be nearing saturation. By continuing to monitor trends with the data over time, and capturing this saturating data point, the fit of the Bass model to this data could be very informative for other jurisdictions wishing to predict outcomes from their own MC programs.

## 5 Conclusion

Data captured by the TGA in the first 5 years following implementation of a MC prescribing regulatory framework in Australia displays rapidly escalating numbers of approvals, particularly since January 2020, and other highly dynamic trends. These data and associated analyses, provide a unique resource that can be drawn upon by researchers, practitioners, and regulators to better understand current clinical practice around MC in Australia, and to identify where research gaps exist relative to prescribing. The analysis presented here shows the utility of such accessible records as the regulatory framework for MC continues to evolve. Other jurisdictions which have initiated, or are looking to initiate, MC schemes might usefully consider the Australian model.

## Data Availability

The raw data supporting the conclusions of this article will be made available by the authors, without undue reservation.

## Footnotes

1 In this study the term “healthcare practitioner” (HCP) refers to the Medical Practitioners and Nurse Practitioners who are authorized to gain access to MC products for these patients. Criteria determining eligibility to prescribe varies across states and territories (RACGP, 2019).
