# Supplementary material for: Medicinal Cannabis Prescribing in Australia: An Analysis of Trends Over the First Five Years
Source: Front Pharmacol. 2022 May 10;13:885655. doi: 10.3389/fphar.2022.885655 (PMC9127064; doi:10.3389/fphar.2022.885655)
Supplement: Supplementary file 1 [file DataSheet1.docx]

Supplementary Material

**Supplementary Figure 1**. Email correspondence to the TGA requesting FOI information.


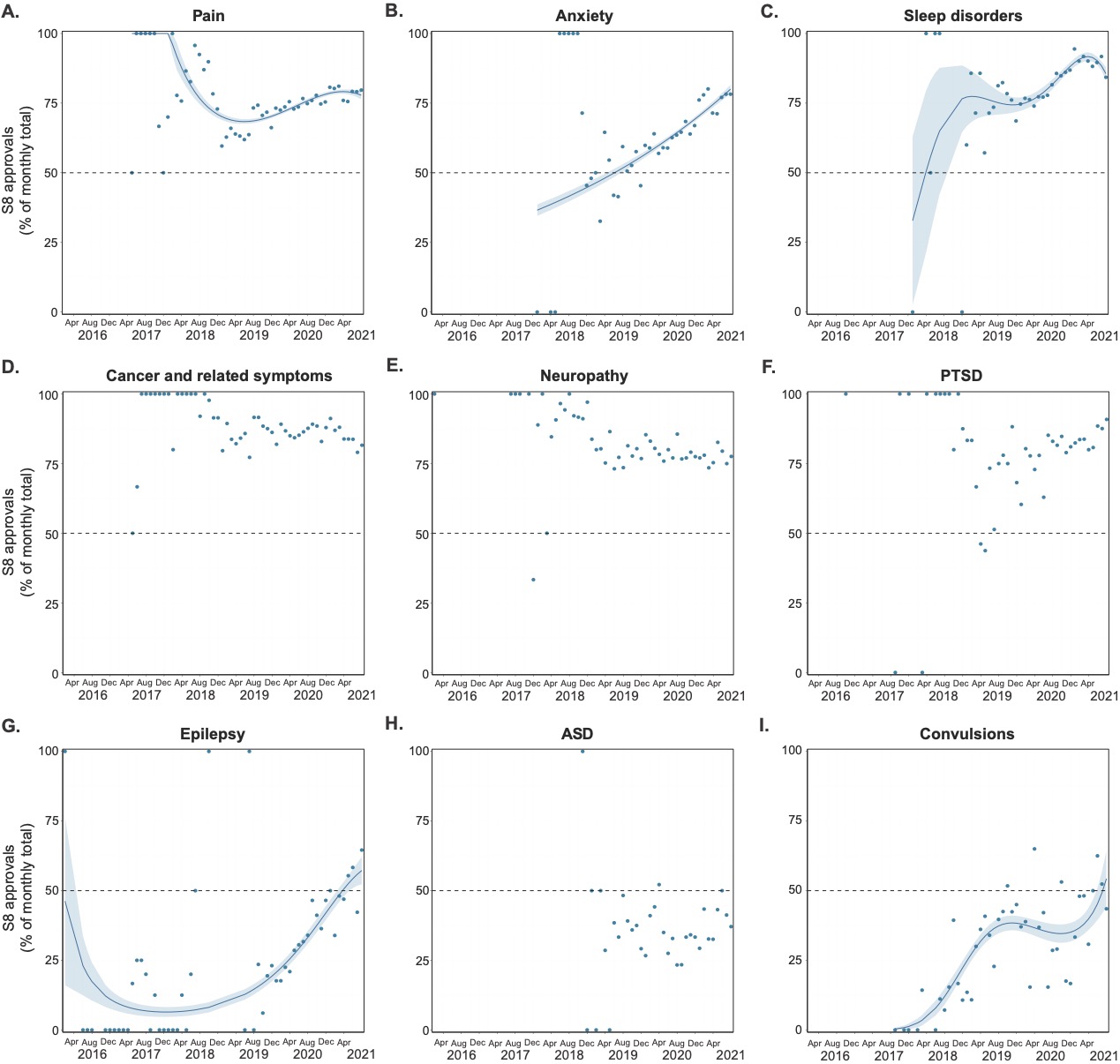


**Supplementary Figure 2.** Approvals for indications categories with >1,000 approvals are predominantly for S8 products. Note that missing datapoints were limited to 18 in this set, and thus the remaining proportion of approvals are almost exclusively for S4 products. The proportion of approvals for pain (A), anxiety (B), sleep disorders (C), cancer and related symptoms (D), neuropathy (E), and PTSD (F) are, on average, predominantly for S8 products, while predominantly S4 for epilepsy (G), ASD (H), and convulsions (L). An increased proportion of S8 approvals over time was observed for anxiety (Negative binomial, 1^st^ degree polynomial, *R^2^* = 0.742), epilepsy (Poisson, 3^rd^ degree polynomial, *R^2^* = 0.656, Δm = 4.482, moderate fit), and convulsions (Poisson, 3^rd^ degree polynomial, *R^2^* = 0.375, Δm = 7.864, weak fit). S8 approvals for cancer, neuropathy, PTSD and ASD were relatively stable over time, while S8 approvals for pain and sleep disorders were stochastic (negative binomial, 3^rd^ degree, *R^2^* = 0.583, Δm = 11.732, moderate fit; Poisson, 4^th^ degree polynomial, *R^2^* = 0.751, Δm = 2.131, respectively).

**Supplementary Table 1.** List of reclassified SAS-B indications according to ICD-10. N refers to the number of approvals in the given indication. SAS-B wording is an unedited list of the unique indications as provided by the TGA.

| Group (N) | Category (*N*, where *N*>1,000) | Indication (ICD-10 code) | *N* | SAS-B wording |
| --- | --- | --- | --- | --- |
| Certain infectious and parasitic diseases (1) | Other sepsis | Sepsis, unspecified (A41.9) | 1 | Sepsis |
| Neoplasms (6,984) | Malignant neoplasm of colon | Colon, unspecified (C18.9) | 1 | Polyposis coli |
|  | Malignant neoplasm of pancreas | Pancreas, unspecified (C25.9) | 2 | Pancreatic cancer |
|  | Malignant neoplasm of bronchus and lung | Bronchus or lung, unspecified (C34.9) | 1 | Lung cancer |
|  | Bone and articular cartilage, unspecified | Bone and articular cartilage, unspecified (C41.9) | 2 | Osteosarcoma |
|  | Malignant neoplasm of breast | Malignant neoplasm of breast, unspecified (C50.9) | 1 | Breast cancer |
|  | Malignant neoplasm of brain | Brain stem (C71.7) | 1 | Brainstem glioblastoma multiforme |
|  |  | Brain, unspecified (C71.9) | 15 | Glioblastoma multiforme |
|  |  |  |  | Glioma |
|  | Multiple myeloma and malignant plasma cell neoplasms | Multiple myeloma (C90.0) | 3 | Multiple myeloma |
|  | Neoplasm of uncertain or unknown behaviour of other and unspecified sites (6,958) | Neoplasm of uncertain or unknown behaviour, unspecified (D48.9) | 6,958 | Cancer |
|  |  |  |  | Cancer Pain |
|  |  |  |  | Cancer pain and symptom management |
|  |  |  |  | Neoplasia |
| Diseases of the blood and blood-forming organs and certain disorders involving the immune mechanism (11) | Other aplastic anaemias | Anaemia, unspecified (D64.9) | 1 | Anaemia |
|  | Sarcoidosis | Sarcoidosis, unspecified (D86.9) | 3 | Sarcoidosis |
|  | Other disorders involving the immune mechanism, not elsewhere classified | Other specified disorders involving the immune mechanism, not elsewhere classified (D89.8) | 7 | Mast cell activation syndrome |
|  |  |  |  | PANDAS - Paediatric autoimmune neuropsychiatric disorder associated with streptococcal infection |
| Endocrine, nutritional and metabolic diseases (6) | Thyrotoxicosis [hyperthyroidism] | Thyrotoxicosis with diffuse goitre (E05.0) | 1 | Graves disease |
|  | Thyroiditis | Autoimmune thyroiditis (E06.3) | 3 | Hashimoto's disease |
|  | Type 1 diabetes mellitus | Type 1 diabetes mellitus without complications (E10.9) | 1 | Insulin dependent diabetes mellitus |
|  | Unspecified diabetes mellitus | Unspecified diabetes mellitus with unspecified complications (E14.8) | 1 | Diabetes mellitus |
| Mental and behavioural disorders (31,315) | Unspecified dementia | Unspecified dementia (F03) | 100 | Dementia |
|  | Mental and behavioural disorders due to use of alcohol | Mental and behavioural disorders due to use of alcohol: dependence syndrome (F10.2) | 1 | Alcohol dependence |
|  | Mental and behavioural disorders due to use of cannabinoids | Mental and behavioural disorders due to use of cannabinoids: unspecified mental and behavioural disorder (F12.9) | 11 | Cannabis Use Disorder |
|  | Schizophrenia | Schizophrenia, unspecified (F20.9) | 16 | Schizophrenia |
|  | Bipolar affective disorder | Bipolar affective disorder, unspecified (F31.9) | 27 | Bipolar disorder |
|  | Recurrent depressive disorder | Recurrent depressive disorder, unspecified (F33.9) | 936 | Depression |
|  |  |  |  | Major depression |
|  |  |  |  | Major depressive disorder |
|  | Other mood [affective] disorders | Other specified mood [affective] disorders (F38.8) | 2 | Premenstrual dysphoric disorder |
|  | Unspecified mood [affective] disorder | Unspecified mood [affective] disorder (F39) | 75 | Mood disorder |
|  | Phobic anxiety disorders | Social phobias (F40.1) | 9 | Social anxiety disorder |
|  | Other anxiety disorders (25,625) | Panic disorder [episodic paroxysmal anxiety] (F41.0) | 5 | Panic disorder |
|  |  | Generalised anxiety disorder (F41.1) | 17 | Generalised anxiety disorder |
|  |  | Anxiety disorder, unspecified (F41.9) | 25,603 | Anxiety |
|  | Obsessive-compulsive disorder | Obsessive-compulsive disorder, unspecified (F42.9) | 3 | Obsessive compulsive disorder |
|  | Reaction to severe stress, and adjustment disorders (2,627) | Post-traumatic stress disorder (F43.1) | 2,627 | Post Traumatic Stress Disorder |
|  |  |  |  | Post Traumatic Stress Disorder (PTSD) |
|  |  |  |  | PTSD - Post-traumatic stress disorder |
|  | Dissociative [conversion] disorders | Dissociative convulsions (F44.5) | 1 | Psychogenic seizures |
|  | Other somatoform disorders | Other somatoform disorders (F45.8) | 1 | Bruxism |
|  | Pervasive developmental disorders (1,262) | Childhood autism (F84.0) | 1,260 | Autism |
|  |  |  |  | Autism spectrum disorder |
|  |  |  |  | Autism spectrum disorder (ASD) |
|  |  | Asperger syndrome (F84.5) | 2 | Asperger syndrome |
|  | Hyperkinetic disorders | Disturbance of activity and attention (F90.0) | 466 | ADHD - Attention deficit disorder with hyperactivity |
|  |  |  |  | Attention deficit hyperactivity disorder (ADHD) |
|  | Tic disorders | Combined vocal and multiple motor tic disorder [de la Tourette] (F95.2) | 103 | Tourette Syndrome |
|  |  |  |  | Tourette's syndrome |
|  | Other behavioural and emotional disorders with onset usually occurring in childhood and adolescence | Unspecified behavioural and emotional disorders with onset usually occurring in childhood and adolescence (F98.9) | 50 | Behaviour disorder |
| Diseases of the nervous system (18,463) | Huntington disease | Huntington disease (G10) | 22 | Huntington chorea |
|  |  |  |  | Huntington's disease |
|  | Hereditary ataxia | Cerebellar ataxia with defective DNA repair (G11.3) | 3 | Ataxia telangiectasia |
|  | Spinal muscular atrophy and related syndromes | Motor neuron disease (G12.2) | 25 | Amyotrophic lateral sclerosis (ALS) |
|  |  |  |  | Motor neuron disease |
|  | Parkinson disease | Parkinson disease (G20) | 754 | Parkinson disease |
|  |  |  |  | Parkinson's disease |
|  |  |  |  | Parkinsonism |
|  |  |  |  | Parkinson’s disease |
|  | Other degenerative diseases of basal ganglia | Progressive supranuclear ophthalmoplegia [Steele-Richardson-Olszewski] (G23.1) | 15 | Progressive supranuclear palsy |
|  |  | Degenerative disease of basal ganglia, unspecified (G23.9) | 8 | Multiple system atrophy |
|  | Dystonia | Drug-induced dystonia (G24.0) | 1 | Tardive dyskinesia |
|  |  | Dystonia, unspecified (G24.9) | 203 | Dyskinesia |
|  |  |  |  | Dystonia |
|  | Other extrapyramidal and movement disorders | Essential tremor (G25.0) | 48 | Essential tremor |
|  |  | Other specified forms of tremor (G25.2) | 2 | Intention tremor |
|  |  |  |  | Primary orthostatic tremor |
|  |  | Myoclonus (G25.3) | 7 | Myoclonic disorder |
|  |  |  |  | Myoclonus |
|  |  | Other specified extrapyramidal and movement disorders (G25.8) | 127 | Restless Legs Syndrome (RLS) |
|  |  |  |  | Stiff Person Syndrome |
|  |  | Extrapyramidal and movement disorder, unspecified (G25.9) | 290 | extrapyramidal syndrome |
|  |  |  |  | Movement disorder |
|  | Alzheimer disease | Alzheimer disease, unspecified (G30.9) | 161 | Alzheimer disease |
|  |  |  |  | Alzheimer's disease |
|  |  |  |  | Alzheimer’s dementia |
|  |  |  |  | Alzheimer’s disease |
|  | Multiple sclerosis | Multiple sclerosis (G35) | 615 | Multiple sclerosis |
|  |  |  |  | Multiple Sclerosis (MS) |
|  |  |  |  | Multiple sclerosis spasm and pain |
|  | Epilepsy (1,349) | Other generalized epilepsy and epileptic syndromes (G40.4) | 16 | Lennox-Gastaut syndrome |
|  |  | Epilepsy, unspecified (G40.9) | 1,333 | Epilepsy |
|  |  |  |  | Refractory epilepsy |
|  | Migraine | Migraine, unspecified (G43.9) | 869 | Migraine |
|  |  |  |  | Migraine (disorder) |
|  | Other headache syndromes | Cluster headache syndrome (G44.0) | 1 | Cluster headache |
|  |  | Other specified headache syndromes (G44.8) | 12 | Chronic headache disorder |
|  | Sleep disorders (9,072) | Disorders of initiating and maintaining sleep [insomnias] (G47.0) | 6,348 | Insomnia |
|  |  | Disorders of excessive somnolence [hypersomnias] (G47.1) | 1 | Hypersomnia |
|  |  | Other sleep disorders (G47.8) | 1 | Parasomnia |
|  |  | Sleep disorder, unspecified (G47.9) | 2,722 | Sleep disorder |
|  | Disorders of trigeminal nerve | Trigeminal neuralgia (G50.0) | 18 | Trigeminal neuralgia |
|  |  | Atypical facial pain (G50.1) | 1 | Left Facial Pain |
|  | Facial nerve disorders | Bell's palsy (G51.0) | 3 | Bell's palsy |
|  | Diseases of other cranial nerves | Disorder of other specified cranial nerves (G52.8) | 1 | Occipital neuralgia |
|  | Cranial nerve disorders in diseases classified elsewhere | Postzoster neuralgia (G53.0) | 1 | Post-herpetic neuralgia |
|  | Inflammatory polyneuropathy | Other inflammatory polyneuropathies (G61.8) | 3 | Chronic inflammatory demyelinating polyneuropathy |
|  | Other polyneuropathies (4,755) | Polyneuropathy, unspecified (G62.9) | 4,755 | Neuropathic pain |
|  |  |  |  | Neuropathy |
|  | Other disorders of peripheral nervous system | Other disorders of peripheral nervous system (G64) | 6 | Peripheral neuropathy |
|  | Myasthenia gravis and other myoneural disorders | Myasthenia gravis (G70.0) | 2 | Myasthenia gravis |
|  | Primary disorders of muscles | Muscular dystrophy (G71.0) | 1 | Muscular dystrophy |
|  |  | Myotonic disorders (G71.1) | 1 | Isaacs syndrome (disorder) |
|  | Cerebral palsy | Cerebral palsy, unspecified (G80.9) | 13 | Cerebral palsy |
|  | Disorders of autonomic nervous system | Complex regional pain syndrome, other and unspecified type (G90.7) | 28 | Complex regional pain syndrome |
|  | Other disorders of brain | Postviral fatigue syndrome (G93.3) | 45 | Chronic fatigue syndrome |
|  |  | Disorder of brain, unspecified (G93.9) | 1 | Neurological disorder |
| Diseases of the eye and adnexa (8) | Other retinal disorders | Degeneration of macula and posterior pole (H35.3) | 3 | Macular degeneration |
|  | Paralytic strabismus | Other paralytic strabismus (H49.8) | 4 | Kearns-Sayre syndrome |
|  | Visual disturbances | Other visual disturbances (H53.8) | 1 | Oscillopsia |
| Diseases of the ear and mastoid process (25) | Disorders of vestibular function | Ménière disease (H81.0) | 3 | Meniere's disease |
|  |  | Vestibular neuronitis (H81.2) | 2 | Vestibular neuronitis |
|  |  | Other disorders of vestibular function (H81.8) | 4 | Mal de debarquement syndrome |
|  | Other disorders of ear, not elsewhere classified | Tinnitus (H93.1) | 16 | Tinnitus |
| Diseases of the circulatory system (1) | Brain stem infarct | Cerebrovascular disease, unspecified (I67.9) | 1 | Brain stem infarct |
| Diseases of the respiratory system (18) | Other chronic obstructive pulmonary disease | Chronic obstructive pulmonary disease, unspecified (J44.9) | 10 | Chronic Obstructive Pulmonary Disease (COPD) |
|  | Asthma | Asthma, unspecified (J45.9) | 6 | Asthma |
|  | Bronchiectasis | Bronchiectasis (J47) | 1 | Bronchiectasis |
|  | Pneumoconiosis due to dust containing silica | Pneumoconiosis due to other dust containing silica (J62.8) | 1 | Silicosis |
| Diseases of the digestive system (371) | Oesophagitis | Oesophagitis (K20) | 3 | Oesophagitis |
|  | Other diseases of oesophagus | Achalasia of cardia (K22.0) | 10 | Achalasia |
|  |  | Barrett oesophagus (K22.7) | 1 | Barrett oesophagus |
|  | Peptic ulcer, site unspecified | Peptic ulcer, site unspecified (K27) | 1 | Ulcer |
|  | Other diseases of stomach and duodenum | Other specified diseases of stomach and duodenum (K31.8) | 6 | Gastroparesis |
|  |  |  |  | Impaired gastric emptying |
|  | Crohn disease | Crohn disease, unspecified (K50.9) | 58 | Crohn's disease |
|  | Ulcerative colitis | Ulcerative colitis, unspecified (K51.9) | 62 | Ulcerative colitis |
|  | Other noninfective gastroenteritis and colitis | Noninfective gastroenteritis and colitis, unspecified (K52.9) | 126 | Inflammatory bowel disease |
|  |  |  |  | Inflammatory Bowel Disease (IBD) |
|  | Irritable bowel syndrome | Other and unspecified irritable bowel syndrome (K58.8) | 104 | Inflammatory Bowel Syndrome (IBS) |
|  |  |  |  | Irritable bowel syndrome |
|  |  |  |  | Irritable Bowel Syndrome (IBS) |
| Diseases of the skin and subcutaneous tissue (47) | Other acantholytic disorders | Transient acantholytic dermatosis [Grover] (L11.1) | 1 | Grover's disease |
|  | Atopic dermatitis | Atopic dermatitis, unspecified (L20.9) | 5 | Atopic dermatitis |
|  | Pruritus | Pruritus, unspecified (L29.9) | 7 | Pruritis |
|  | Other dermatitis | Dermatitis, unspecified (L30.9) | 17 | Dermatitis |
|  |  |  |  | Eczema |
|  | Psoriasis | Psoriasis, unspecified (L40.9) | 12 | Psoriasis |
|  | Other papulosquamous disorders | Pityriasis rubra pilaris (L44.0) | 1 | Pityriasis rubra pilaris |
|  | Rosacea | Rosacea, unspecified (L71.9) | 3 | Rosacea |
|  | Vasculitis limited to skin, not elsewhere classified | Vasculitis limited to skin, unspecified (L95.9) | 1 | Vasculitis |
| Diseases of the musculoskeletal system and connective tissue (1,161) | Other rheumatoid arthritis | Rheumatoid arthritis, unspecified (M06.9) | 54 | Rheumatoid arthritis |
|  | Psoriatic and enteropathic arthropathies | Other psoriatic arthropathies (M07.3) | 3 | Psoriatic arthritis |
|  | Other arthritis | Arthritis, unspecified (M13.9) | 193 | Arthritis |
|  | Other arthrosis | Arthrosis, unspecified (M19.9) | 45 | Osteoarthritis |
|  | Other joint disorders, not elsewhere classified | Pain in joint (M25.5) | 2 | Joint pain |
|  | Systemic lupus erythematosus | Systemic lupus erythematosus, unspecified (M32.9) | 3 | Lupus |
|  | Systemic sclerosis | Systemic sclerosis, unspecified (M34.9) | 2 | Scleroderma |
|  | Other deforming dorsopathies | Torticollis (M43.6) | 1 | Torticollis |
|  | Spondylosis | Other spondylosis (M47.8) | 1 | Cervical spondylosis |
|  | Other soft tissue disorders, not elsewhere classified | Myalgia (M79.1) | 4 | Muscle pain |
|  |  | Neuralgia and neuritis, unspecified (M79.2) | 6 | Neuralgia |
|  |  | Fibromyalgia (M79.7) | 845 | Fibromyalgia |
|  | Osteoporosis without pathological fracture | Osteoporosis, unspecified (M81.9) | 2 | Osteoporosis |
| Diseases of the genitourinary system (15) | Other disorders of penis | Disorder of penis, unspecified (N48.9) | 1 | Penile irritation |
|  | Endometriosis | Endometriosis, unspecified (N80.9) | 14 | Endometriosis |
| Congenital malformations, deformations and chromosomal abnormalities (4) | Congenital malformations of the musculoskeletal system, not elsewhere classified | Ehlers-Danlos syndrome (Q79.6) | 2 | Ehlers-Danlos syndrome |
|  | Other congenital malformations of skin | Mastocytosis (Q82.2) | 2 | Mastocytosis |
| Symptoms, signs and abnormal clinical and laboratory findings, not elsewhere classified (100,744) | Cough | Cough (R05) | 1 | Cough |
|  | Abnormalities of breathing | Dyspnoea (R06.0) | 3 | Dyspnea |
|  | Nausea and vomiting | Nausea and vomiting (R11) | 766 | Chemotherapy Induced Nausea and Vomitting (CINV) |
|  |  |  |  | Chemotherapy induced vomiting |
|  |  |  |  | Chronic Nausea |
|  |  |  |  | Nausea |
|  |  |  |  | Nausea and vomiting |
|  |  |  |  | Refractory nausea and vomiting |
|  | Abnormal involuntary movements | Tremor, unspecified (R25.1) | 173 | Tremor |
|  |  | Cramp and spasm (R25.2) | 608 | Muscle spasm |
|  |  |  |  | Muscle spasticity |
|  |  |  |  | Spasticity |
|  |  |  |  | Trismus |
|  |  | Other and unspecified abnormal involuntary movements (R25.8) | 2 | Involuntary movement disorder |
|  | Other lack of coordination | Ataxia, unspecified (R27.0) | 2 | Ataxia |
|  | Other symptoms and signs involving the nervous ad musculoskeletal systems | Other and unspecified symptoms and signs involving the nervous and musculoskeletal systems (R29.8) | 16 | Muscle rigidity |
|  | Other symptoms and signs involving cognitive functions and awareness | Other amnesia (R41.3) | 1 | Memory loss |
|  |  | Other and unspecified symptoms and signs involving cognitive functions and awareness (R41.8) | 3 | Cognitive decline |
|  |  |  |  | Intellectual impairment |
|  | Dizziness and giddiness | Dizziness and giddiness (R42) | 6 | Dizziness |
|  |  |  |  | Dysequilibrium |
|  |  |  |  | Vertigo |
|  | Symptoms and signs involving emotional state | Restlessness and agitation (R45.1) | 9 | Agitation |
|  |  | Hostility (R45.5) | 13 | Aggressive behaviour |
|  | General symptoms and signs | Headache (R51) | 33 | Headache |
|  | Pain, not elsewhere classified (97,400) | Other chronic pain (R52.2) | 97,288 | Chronic pain |
|  |  | Pain, unspecified (R52.9) | 112 | Pain |
|  | Malaise and fatigue | Malaise and fatigue (R53) | 7 | Fatigue |
|  | Convulsions, not elsewhere classified (1,229) | Other and unspecified convulsions (R56.8) | 1,229 | Seizure management |
|  | Hyperhidrosis | Hyperhidrosis, unspecified (R61.9) | 2 | Hyperhidrosis |
|  | Symptoms and signs concerning food and fluid intake | Anorexia (R63.0) | 456 | Anorexia |
|  |  |  |  | Appetite control |
|  | Cachexia | Cachexia (R64) | 11 | Cachexia |
|  | Systemic Inflammatory Response Syndrome [SIRS] | Systemic Inflammatory Response Syndrome, unspecified (R65.9) | 1 | SIRS - Systemic inflammatory response syndrome score |
|  | Elevated blood glucose level | Hyperglycaemia, unspecified (R73.9) | 1 | Blood glucose management |
|  | Abnormal findings in specimens from other organs, systems and tissues | Abnormal findings in specimens from other organs, systems and tissues: abnormal histological findings (R89.7) | 1 | Dysplasia |
| Factors influencing health status and contact with health services (476) | Care involving use of rehabilitation procedures | Care involving use of other rehabilitation procedures (Z50.8) | 1 | Smoking cessation assistance |
|  | Other medical care | Palliative care (Z51.5) | 475 | Palliative care |
| Inadequate information (13) | - | - | 1 | Atrophy |
|  |  |  | 1 | Autoimmune disorder |
|  |  |  | 1 | Genetic disease |
|  |  |  | 1 | Hypersensitivity |
|  |  |  | 9 | Inflammation |

**Supplementary Table 2.** List of reclassified product formats. Product presentations supplied within the TGA data had varied wordings, and were re-classified based on simplified taxonomy, presented here in alphabetical order. N = number of total applications per product format, S4 = Schedule 4. S8 = Schedule 8.

| Product Format | N | S4 (%) | S8 (%) | Product Presentation (Provided wording) |
| --- | --- | --- | --- | --- |
| Capsules | 3,976 | 57.6% | 42.4% | Capsule |
|  |  |  |  | Capsules |
|  |  |  |  | Oral Capsule |
|  |  |  |  | Soft Gelatin Capsules |
|  |  |  |  | Softgel Capsule |
| Crystal | 182 | 100.0% | 0.0% | Crystals |
| Flower | 37,727 | 0.0% | 100.0% | Bud |
|  |  |  |  | Cannabis flos |
|  |  |  |  | cannabis flos granulate |
|  |  |  |  | Cannabis Granulate |
|  |  |  |  | Cannabis Oil |
|  |  |  |  | Dried Cannabis |
|  |  |  |  | Dried cannabis bud |
|  |  |  |  | Dried Flower |
|  |  |  |  | Dried Herb |
|  |  |  |  | Flos |
|  |  |  |  | Herb,Dried |
|  |  |  |  | inhalation |
|  |  |  |  | Vaporisation |
|  |  |  |  | Whole Dried Cannabis Flower |
|  |  |  |  | Whole Flower |
| Lozenge | 135 | 100.0% | 0.0% | Lozenge |
| Oil | 11,2101 | 31.8% | 68.2% | Drops |
|  |  |  |  | Liquid |
|  |  |  |  | Oil |
|  |  |  |  | Oil Solution |
|  |  |  |  | Oil Solution (30 mL Bottle) |
|  |  |  |  | Oral Liquid |
|  |  |  |  | Oral Solution |
|  |  |  |  | Oral Solution |
|  |  |  |  | Oral Tincture |
|  |  |  |  | Solution |
| Spray | 4,583 | 21.0% | 79.0% | (Nabiximols) |
|  |  |  |  | Oral Spray Solution |
|  |  |  |  | Pressurised Metered Dose Inhaler |
|  |  |  |  | Spray |
| Tablet | 134 | 100.0% | 0.0% | Chew Tablets |
|  |  |  |  | Medicated Chew |
|  |  |  |  | Tablet |
| Topical | 215 | 94.0% | 6.0% | Topical Gel |
|  |  |  |  | Topical Preparation |
|  |  |  |  | Transdermal Gel |
|  |  |  |  | Transdermal patch |
|  |  |  |  | Vaginal Cream |
| Wafer | 594 | 100.0% | 0.0% | Wafer |
| Unknown | 18 | 0.0% | 100.0% | Product |

**Supplementary Table 3.** Contribution to variance relating to Figure 7A.

| Variable 1 | Dim 1 (%) | Dim 2 (%) | Variable 2 | Dim 1 (%) | Dim 2 (%) |
| --- | --- | --- | --- | --- | --- |
| Anxiety | 1.542 | 53.995 | **<18** | 92.089 | 5.343 |
| Cancer and related symptoms | 0.707 | 17.54 | **18-30** | 2.305 | 34.878 |
| ASD | 60.905 | 5.014 | **31-37** | 0.000 | 14.179 |
| Epilepsy | 19.383 | 0.192 | **38-44** | 0.263 | 3.542 |
| Neuropathy | 0.404 | 4.043 | **45-52** | 0.579 | 0.166 |
| Pain | 4.288 | 8.896 | **53-60** | 1.131 | 4.745 |
| PTSD | 0.001 | 3.481 | **61-71** | 1.613 | 16.037 |
| Convulsions | 12.770 | 0.029 | **>71** | 2.019 | 21.112 |
| Sleep disorders | 0.000 | 6.810 |  |  |  |

**Supplementary Table 4.** Contribution to variance relating to Figure 7B.

| Variable 1 | Dim 1 (%) | Dim 2 (%) | Variable 2 | Dim 1 (%) | Dim 2 (%) |
| --- | --- | --- | --- | --- | --- |
| Anxiety | 3.931 | 9.894 | **Capsules** | 0.264 | 0.416 |
| Cancer and related symptoms | 3.196 | 18.804 | **Crystal** | 0.029 | 0.041 |
| ASD | 7.488 | 0.095 | **Flower** | 24.745 | 47.145 |
| Epilepsy | 0.375 | 1.792 | **Lozenge** | 0.338 | 0.059 |
| Neuropathy | 1.828 | 9.139 | **Oil** | 4.638 | 11.356 |
| Pain | 0.009 | 4.522 | **Spray** | 1.113 | 11.078 |
| PTSD | 0.930 | 1.950 | **Tablet** | 0.273 | 0.040 |
| Convulsions | 70.930 | 22.693 | **Topical** | 68.535 | 29.862 |
| Sleep disorders | 11.314 | 32.111 | **Wafer** | 0.066 | 0.003 |

**Supplementary Table 5.** Contribution to variance relating to Figure 7C.

| Variable 1 | Dim 1 (%) | Dim 2 (%) | Variable 2 | Dim 1 (%) | Dim 2 (%) |
| --- | --- | --- | --- | --- | --- |
| <18 | 4.467 | 92.536 | **Capsules** | 2.994 | 2.260 |
| 18-30 | 23.702 | 1.055 | **Crystal** | 0.571 | 0.112 |
| 31-37 | 15.005 | 0.000 | **Flower** | 73.801 | 0.256 |
| 38-44 | 7.377 | 0.342 | **Lozenge** | 0.137 | 17.793 |
| 45-52 | 0.027 | 0.046 | **Oil** | 17.580 | 0.380 |
| 53-60 | 2.279 | 0.461 | **Spray** | 4.333 | 8.706 |
| 61-71 | 16.577 | 1.033 | **Tablet** | 0.089 | 8.578 |
| >71 | 30.566 | 4.527 | **Topical** | 0.000 | 61.483 |
|  |  |  | **Wafer** | 0.495 | 0.432 |
